# Long-term efficacy and safety of subcutaneous pasireotide alone or in combination with cabergoline in Cushing’s disease

**DOI:** 10.3389/fendo.2023.1165681

**Published:** 2023-10-09

**Authors:** Richard A. Feelders, Maria Fleseriu, Pinar Kadioglu, Marie Bex, Deyanira González-Devia, Cesar Luiz Boguszewski, Dilek Gogas Yavuz, Heather Patino, Alberto M. Pedroncelli, Ricardo Maamari, Arghya Chattopadhyay, Beverly M. K. Biller, Rosario Pivonello

**Affiliations:** ^1^ Department of Internal Medicine, Division of Endocrinology, Erasmus Medical Center, Rotterdam, Netherlands; ^2^ Pituitary Center, Departments of Medicine and Neurological Surgery, Oregon Health & Science University, Portland, OR, United States; ^3^ Division of Endocrinology, Metabolism and Diabetes, Cerrahpasa Medical Faculty, Istanbul University - Cerrahpasa, Istanbul, Türkiye; ^4^ Department of Endocrinology, University Hospitals Leuven, Leuven, Belgium; ^5^ Departamento de Medicina Interna, Sección de Endocrinologia, Hospital Universitario Fundación Santa Fé de Bogotá, Bogota, Colombia; ^6^ Department of Internal Medicine, Endocrine Division (SEMPR), Federal University of Paraná, Curitiba, Parana, Brazil; ^7^ Section of Endocrinology and Metabolism, Marmara University School of Medicine, Department of Internal Medicine, Division of Endocrinology and Metabolism, Istanbul, Türkiye; ^8^ Global Medical Affairs, Novartis Pharmaceuticals Corporation, East Hanover, NJ, United States; ^9^ Recordati AG, Basel, Switzerland; ^10^ Global Medical Affairs, Novartis Pharma AG, Basel, Switzerland; ^11^ Global Medical Affairs, Novartis Healthcare Private Limited, Hyderabad, Telangana, India; ^12^ Neuroendocrine & Pituitary Tumor Clinical Center, Massachusetts General Hospital, Boston, MA, United States; ^13^ Dipartimento di Medicina Clinica e Chirurgia, Sezione di Endocrinologia, Università Federico II di Napoli, Naples, Italy

**Keywords:** somatostatin, pasireotide, cabergoline, Cushing’s disease, hypercortisolism

## Abstract

**Objective:**

This study evaluated short- and long-term efficacy and safety of the second-generation somatostatin receptor ligand pasireotide alone or in combination with dopamine agonist cabergoline in patients with Cushing’s disease (CD).

**Study design:**

This is an open-label, multicenter, non-comparative, Phase II study comprising 35-week core phase and an optional extension phase. All patients started with pasireotide, and cabergoline was added if cortisol remained elevated. Eligible patients had active CD, with or without prior surgery, were pasireotide naïve at screening or had discontinued pasireotide for reasons other than safety. Primary endpoint was proportion of patients with a mean urinary free cortisol (mUFC) level not exceeding the upper limit of normal (ULN) at week 35 with missing data imputed using last available post-baseline assessments.

**Results:**

Of 68 patients enrolled, 26 (38.2%) received pasireotide monotherapy and 42 (61.8%) received pasireotide plus cabergoline during the core phase. Thirty-four patients (50.0%; 95% CI 37.6–62.4) achieved the primary endpoint, of whom 17 (50.0%) received pasireotide monotherapy and 17 (50.0%) received combination therapy. Proportion of patients with mUFC control remained stable during the extension phase up to week 99. Treatment with either mono or combination therapy provided sustained improvements in clinical symptoms of hypercortisolism up to week 99. Hyperglycemia and nausea (51.5% each), diarrhea (44.1%) and cholelithiasis (33.8%) were the most frequent adverse events.

**Conclusion:**

Addition of cabergoline in patients with persistently elevated mUFC on maximum tolerated doses of pasireotide is an effective and well-tolerated long-term strategy for enhancing control of hypercortisolism in some CD patients.

**Clinical trial registration:**

https://clinicaltrials.gov/ct2/show/NCT01915303, identifier NCT01915303.

## 1 Introduction

Cushing’s disease (CD) is a rare condition arising from chronic overproduction of cortisol, secondary to an adrenocorticotropic hormone (ACTH)-secreting pituitary tumor (1). Untreated hypercortisolism results in substantial multisystem morbidity, impaired quality of life (QoL) and premature mortality (1–4).

Pasireotide is a second-generation, multireceptor-targeted somatostatin receptor ligand (SRLs), with affinity for 4 of the 5 known somatostatin receptor subtypes (SSTRs) (5) and is approved for the treatment of patients with CD for whom surgery has failed or is not an option (6). Phase III trials of pasireotide monotherapy have shown sustained biochemical and clinical benefits up to 5 years (6–9). These benefits are also reflected in real-world evidence (10).

Cabergoline, a potent dopamine agonist with high affinity for dopamine type 2 receptors (D2), is commonly used off-label for the treatment of CD (2). Small, retrospective, non-randomized studies have demonstrated long-term urinary free cortisol (UFC) control (24−;60 months) in 23−;40% of patients with CD, especially those with mild hypercortisolism (11–13). A meta-analysis of individual patient data from six observational studies (n=124) reported normalization of mean UFC (mUFC) levels in 34% of patients (14, 15). However, a short prospective study on cabergoline monotherapy showed a limited value in controlling UFC, possibly linked to short duration (16). As most corticotropinomas co­express SSTR5 and D2, combining pasireotide and cabergoline in a stepwise approach could potentially improve efficacy with achieving more rapid biochemical control (17), a premise supported by results from an 80-day pilot study of 17 patients with CD treated with cabergoline- pasireotide combination, and low-dose ketoconazole (in case of lack of complete control with the two-drug combination) (18).

The current study aims to report the efficacy and safety of prolonged treatment with pasireotide alone or in combination with cabergoline from the largest prospective, multicentre study to date of a pituitary-targeting combination treatment regimen in patients with CD (NCT01915303).

## 2 Materials and methods

### 2.1 Patients

Adults (≥18 years) with a confirmed diagnosis of CD or *de novo* CD, if they were not candidates for surgery or refused surgery were enrolled. Cushing’s disease was defined by a mean 24-hour (24h) UFC level greater than the upper limit of normal (ULN, 137.95 nmol/24h), calculated from three 24h samples collected within 2 weeks; a morning plasma ACTH level within or above the normal range; and a confirmed pituitary source of Cushing’s syndrome, determined by MRI confirmation of pituitary adenoma >6mm or inferior petrosal sinus sampling (IPSS) gradient >3 after CRH stimulation (or >2 if IPSS without CRH stimulation) for those patients with a tumor ≤6mm. For patients who had prior pituitary surgery, histopathology confirming an ACTH staining adenoma was considered confirmatory of CD. Key exclusion criteria included optic chiasm compression requiring surgery, poorly controlled diabetes (glycated hemoglobin [HbA_1c_] >8%) and having risk factors for torsades de pointes (for further details, see the **Supplementary Appendix**).

### 2.2 Study design

This was a single-arm, open-label, multicenter, non-comparative, Phase II study. After 4 weeks of screening, patients were treated in a stepwise approach during the core phase. Patients received subcutaneous pasireotide 0.6 mg twice daily (bid) for 8 weeks. Patients with a mUFC level exceeding ULN after 8 weeks received pasireotide 0.9 mg bid for another 8 weeks. If mUFC level remained elevated with pasireotide 0.9 mg bid, oral cabergoline 0.5 mg once daily (qd) was added for 8 weeks and could be increased to 1.0 mg qd for another 8 weeks (**Supplementary Figure S1**). After 35 weeks of treatment in the core phase, patients could enter the extension phase of the trial. Addition or titration of cabergoline during the extension phase was at the discretion of investigators.

Collection of extension data commenced from week 43, and patients continued their current study treatment up to study end (4 September 2019; date of last patient visit), week 257. Data beyond week 99 are not reported here because of small patient numbers.

### 2.3 End points and assessments

The primary endpoint of the study was the proportion of patients with mUFC ≤ULN at week 35. Secondary endpoints (reported at 4-week intervals up to week 35 and 8-week intervals from week 43 to the date of the last patient visit) included changes from baseline in mUFC, plasma ACTH, serum cortisol, total cholesterol, and clinical signs (systolic/diastolic blood pressure, body mass index (BMI), weight, waist circumference, facial rubor, hirsutism, striae, supraclavicular and dorsal fat pads) and symptoms (CushingQoL). Treatment escape was defined as an increase in one UFC above the normal range during follow-up of complete responders (14). Cushing Quality of Life Questionnaire (CushingQoL) (19) scores were reported up to week 35 only. Details on the safety assessments are provided in the **Supplementary Appendix**.

### 2.4 Statistical analyses

No formal hypothesis testing was performed because of the exploratory design of the study. Efficacy analyses were conducted on full analysis set, i.e., all patients to whom study treatment was assigned. Safety analyses were conducted on all patients who received ≥1 dose of pasireotide per day during the study. For patients with missing mUFC value at week 35, including those who discontinued, the last available assessment was carried forward. Details on the *post hoc* analyses and sample size estimation is provided in the **Supplementary Appendix**. Enrolled patients, who were observed for failed inclusion or exclusion criteria during the monitoring visits, were classified under protocol deviation. However, patients with no safety concerns were allowed to continue in the study and included in the full analysis set as intention to treat – assessing the study outcome, while some patients were excluded from the per protocol analysis.

## 3 Results

### 3.1 Study population

**Figure 1:**
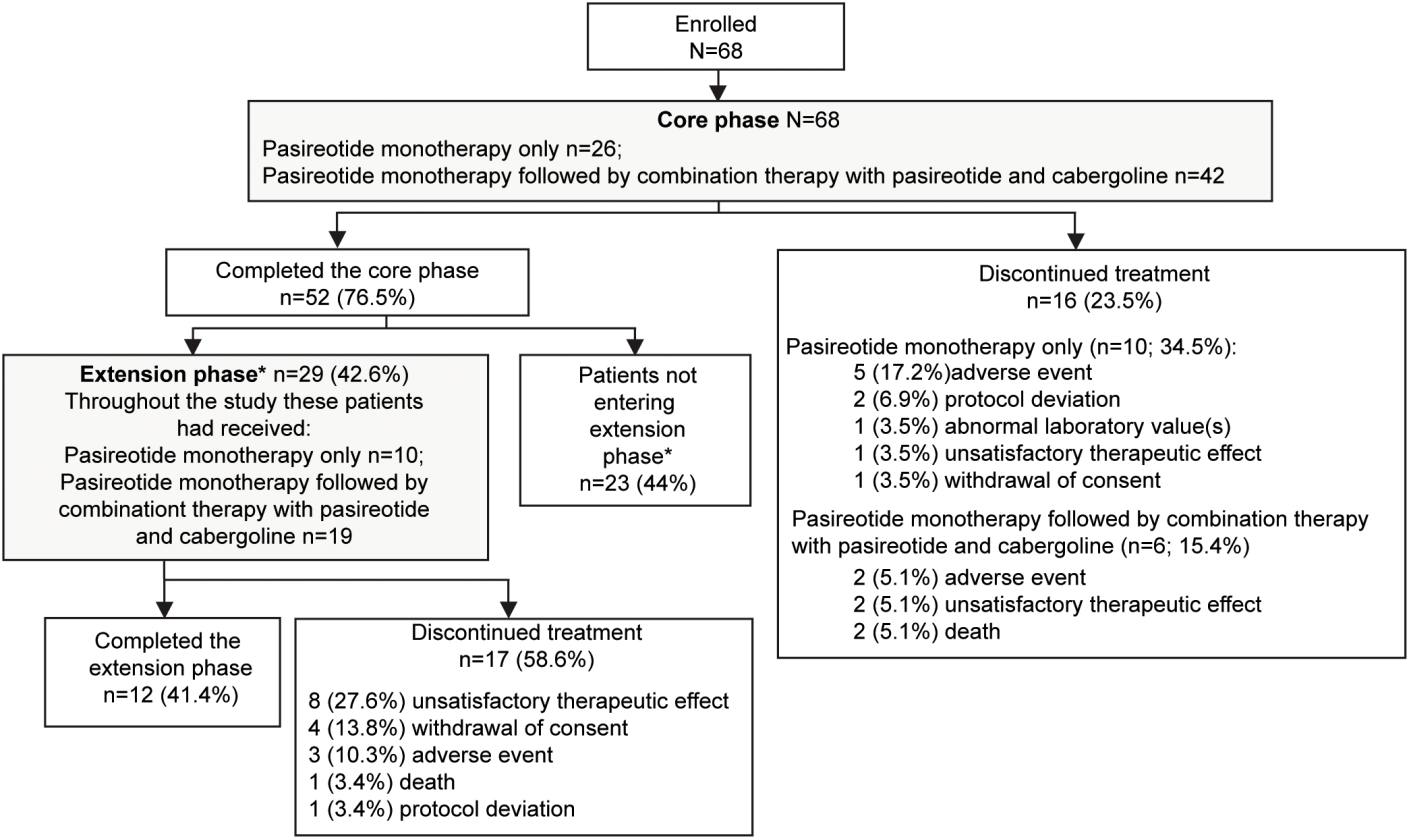
Patient disposition. *If the study drugs were locally available at the end of the core phase, patients could switch over to the commercial supply and exit the extension phase. Only in countries where the drug was not locally available were patients given the option to enter the extension phase. Percentage for patients not entering the extension phase was calculated from the total number of patients enrolled in the study.

A total of 68 patients were enrolled in the study. At baseline, 66 (97.1%) patients were pasireotide naïve, while 2 (2.9%) were treated with pasireotide previously with 4 weeks of washout period prior to screening (**Table 1**). Of 68 patients received treatment during the core phase, 26 (38.2%) received pasireotide monotherapy and 42 (61.8%) received combination therapy. Fifty-two (76.5%) patients completed the 35-week core phase while 16 (23.5%) discontinued (**Figure 1**). All 68 patients were included in the full analysis set based on the intention to treat (ITT) principle. One of the protocol deviations observed during the study, was inclusion of 3 patients with normal mUFC value at screening visit (baseline) and assigning a treatment. The deviation category for the 3 patients was ‘failed inclusion criteria’ with screening mUFC value ≤ULN (137.95 nmol/24h) or mUFC calculated using <3 UFC values or 2 out of 3 UFC values ≤ULN. One of these patients (baseline mUFC 37.37 nmol/24h ≤ULN) was discontinued from the study at Week 2 and due to lack of post-baseline mUFC assessment, was classified ‘non-responder’ at Week 35 assessment. The 2^nd^ patient’s baseline mUFC value of 135.20 nmol/24h was close to ULN (137.95 nmol/24h) and was rescreened. Based on the rescreened mUFC value 306.5 nmol/24h, this patient was included in study, and the mUFC at Week 35 was 192.30 nmol/24h (non-responder at Week 35 assessment). For all study assessments, the scheduled screening visit’s first mUFC value (≤ULN) was used as baseline value. The 3^rd^ patient (baseline mUFC value 131.77 nmol/24h) was discontinued from the study at Week 26 and was also observed for non-compliant schedule visit and medication dosages. The mUFC value recorded at Week 26 (88.95 nmol/24h) was ≤ULN and this last observation was carried forward to Week 35. Hence, the patient was classified ‘responder’, leaving one patient included in the study as responder as a protocol deviation.

**Table 1:** Patient demographics and baseline characteristics.

	N=68
Age, years	
Mean (SD)	41.4 (13.9)
Median (range)	40.5 (19–79)
Sex, n (%)	
Male	8 (11.8)
Female	60 (88.2)
Cushing’s disease status, n (%)	
*De novo*	10 (14.7)
Persistent/recurrent*	58 (85.3)
Prior pituitary surgery, n (%)	54 (79.4)
Prior pituitary irradiation, n (%)	13 (19.1)
Time since diagnosis, months^†^	
Mean (SD)	56.8 (47.0)
Median (range)	41.6 (4–199)
Prior exposure to study drugs, n (%)	
Naïve to cabergoline	45 (66.2)
Naïve to pasireotide	66 (97.1)
Previously treated with cabergoline	23 (33.8)
Previously treated with pasireotide	2 (2.9)
**UFC (nmol/24h), Mean (SD), x ULN**	501.6 (488.66), 3.64
mUFC category, n (%)^‡^	
Mild hypercortisolism	23 (33.8)
Moderate hypercortisolism	30 (44.1)
Severe hypercortisolism	12 (17.6)

*Persistent or recurrent disease refers to patients with previous surgery or on medication who did not respond or experienced escape. **^†^** Time since diagnosis is based on available data for 43 patients; **^‡^** 3/68 (4.4%) patients had mUFC ≤ULN at baseline. Hypercortisolism: mild hypercortisolism, mUFC >1.0–<2.0 x ULN; moderate hypercortisolism, mUFC 2.0–5.0 x ULN; severe hypercortisolism, mUFC >5.0 x ULN. ULN; 137.95 nmol/24h.

mUFC, mean urine free cortisol; SD, standard deviation; ULN, upper limit of normal.

Twenty-nine (42.6%) patients continued treatment in the extension phase; 10 (34.5%) received pasireotide monotherapy and 19 (65.5%) received combination therapy. Twelve (41.4%) patients completed the extension phase, while 17 (58.6%) discontinued treatment before study end, most commonly for unsatisfactory therapeutic effect (n=8). The most common reason for discontinuation was adverse events (AEs): 5 (17.2%) patients with pasireotide monotherapy and 2 (5.1%) patients with combination therapy.

### 3.2 Efficacy: biochemical response

**Figure 2:**
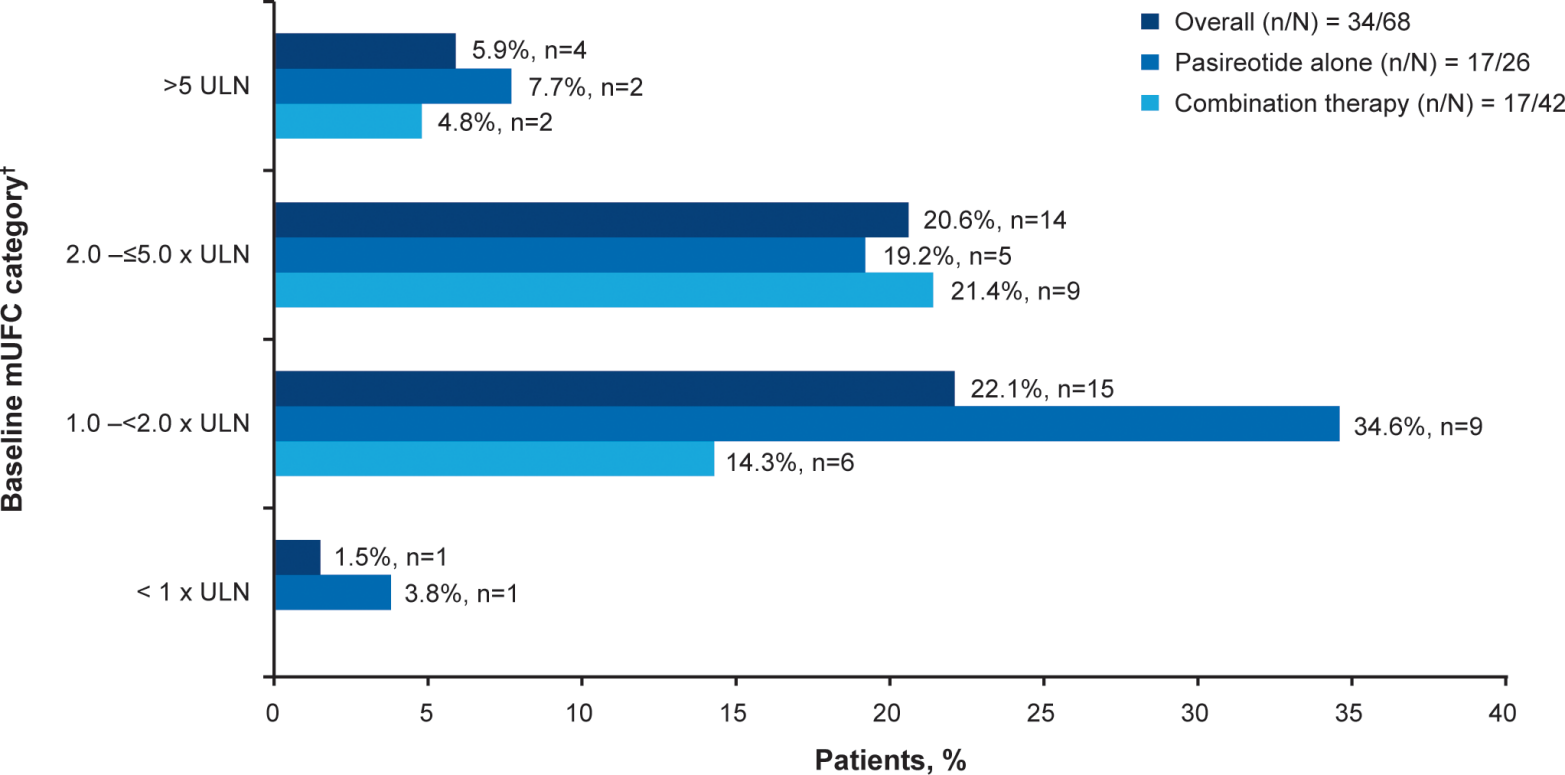
Patients achieving mUFC ≤ULN at week 35. ^†^At baseline there were 23 patients with mild, 30 with moderate and 12 with severe hypercortisolism. mUFC, mean urine free cortisol; ULN, upper limit of normal.

Overall, 34/68 (50.0%; 95% CI 37.6–62.4) patients achieved the primary endpoint, of whom 17 (50.0%) were receiving pasireotide monotherapy and 17 (50.0%) were receiving combination therapy. Patients with mild hypercortisolism (mUFC 1.0–<2.0 x ULN) at baseline were more likely to respond to both pasireotide monotherapy and combination therapy (n=15; 22.1%, **Figure 2**). Seven of 17 patients in the pasireotide monotherapy group met the primary endpoint based on their last available assessment prior to week 35. Even if the 3 patients who had mUFC ≤ULN at baseline were excluded from the primary analysis, 33/65 (50.7%; 95% CI 38.1–63.4) patients would have achieved the primary endpoint. The results are similar to the original analysis (34/68 (50.0%; 95% CI 37.6–62.4) based on the full analysis set.

For the overall study population (n=68), mUFC rapidly decreased from 501.6 nmol/24h (3.6 x ULN; SD: 488.66 nmol/24h) to 242.1 nmol/24h (1.8 x ULN; SD: 203.47 nmol/24h) at week 4 and mUFC remained below baseline levels up to week 35 (184.8 nmol/24h; 1.3 x ULN; SD:140.13 nmol/24h). For patients who received pasireotide monotherapy (n=26), mUFC( ± SD) decreased from baseline (442.1± 557.13 nmol/24h [n=26]; 3.2 x ULN) to week 35 (136.6 ± 127.77 nmol/24h [n=14]; 1 x ULN) and at the end of the study (111.2 ± 40.39 nmol/24h [n=5]; 0.8 x ULN) using the last-observation-carried-forward (LOCF). For those who did not normalize on pasireotide monotherapy (n=42), mUFC ( ± SD) decreased from baseline, i.e., last observation before starting cabergoline (280.20 ± 129.03 nmol/24h [n=40]; 2.0 x ULN) to week 35 (206.6 ± 141.96 nmol/24h [n=31]; 1.5 x ULN) and at the end of the study (219.60 ± 83.78 nmol/24h [n=7]; 1.6 x ULN) using the LOCF. During the core phase, mean serum cortisol decreased from 738.6 nmol/L (1.3 x ULN) at baseline to 538.2 nmol/L (0.95 x ULN) and ACTH levels from 16.3 pmol/L (2.7 x ULN) to 11.0 pmol/L (1.8 x ULN) at week 35.

**Figure 3:**
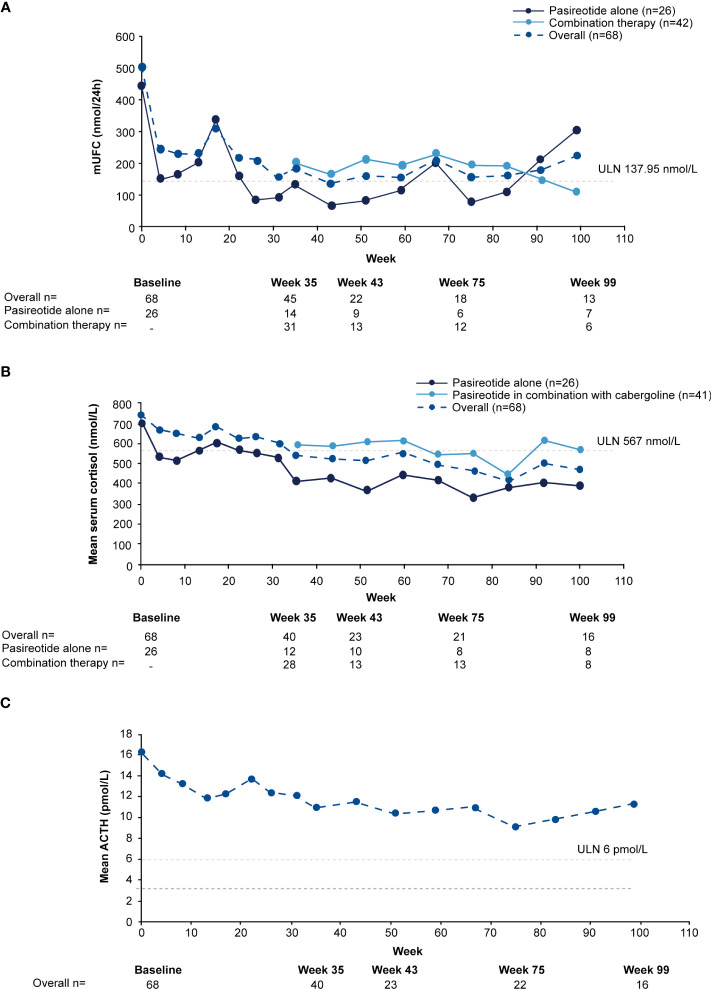
Mean actual change over time in **(A)** mUFC **(B)** serum cortisol, and **(C)** ACTH. ACTH, adrenocorticotropic hormone; mUFC, mean urine free cortisol; ULN, upper limit of normal .

During the extension phase, 25 patients had a mUFC assessment; of whom 12 (48%) had a mUFC ≤ULN at the end of the extension phase. During the extension phase, mUFC levels decreased slightly and fluctuated above and below the ULN up to the week 139 (**Figure 3A**), while mean serum cortisol remained below ULN (404 nmol/L; **Figure 3B**) and ACTH levels fluctuated from 8.2 pmol/L to 11.5 pmol/L) and remained above the ULN value (**Figure 3C**).

Twenty-one of 38 (55%) patients achieved control with combination therapy at some point during the core or extension study, of whom 13 (62%) experienced escape (at least one UFC >ULN after previous control). The time to achieve control after starting cabergoline ranged from 14−;343 days. Notably, one patient received pasireotide 0.6 mg bid initially, dose increased to 0.9 mg bid at Week 17, followed by addition of cabergoline 0.5 mg od at Week 31. The patient achieved biochemical control (mUFC value of 120.15 nmol/24h) on the same day of the start of combination therapy. Clinically it is highly unlikely that biochemical control was achieved with single dose of cabergoline administration. Therefore, it could be considered that normalization was achieved while receiving pasireotide monotherapy. Also, the physician might have prescribed combination therapy before receiving the mUFC value of the (urinary) sample delivered on the morning of combination therapy initiation (while the patient was still on monotherapy). The patient continued combination therapy and maintained biochemical control up to Week 35 and beyond. Furthermore, at Week 59 the cabergoline dose was increased to 1.0 mg/day due to mUFC >ULN at previous visit (Week 51). The patient remained on pasireotide 0.9 mg bid/cabergoline 1.0 mg od combination therapy until the study end.

The median time to escape after achieving control with the addition of cabergoline was 58 days (range 28−;344). 10/13patients regained biochemical control with combination therapy. No patients on pasireotide alone experienced escape, probably due to the short observation time.

### 3.3 Clinical signs and symptoms of CD

Relative to baseline, pasireotide monotherapy was accompanied by reductions in median blood pressure, weight, BMI, waist circumference, and total cholesterol. Overall improvement in clinical measures persisted over time (**Supplementary Table S1**). Clinical improvements were also seen following the addition of cabergoline, particularly for hirsutism (**Supplementary Figures S2**, **S3**).

Mean( ± SD) standardized CushingQoL score was 41.6(± 20.2) at baseline and increased to 47.6(± 20.8) at week 35 (**Supplementary Table S2**), indicating improvements in patients’ QoL (19).

### 3.4 Safety and tolerability

Median duration of exposure to pasireotide was 35.0 weeks (range 0−;268), with a median dose of 1.53 mg/day (range 0.29−;1.80). Median duration of exposure to cabergoline was 16.9 weeks (range 1−;215), with a median dose of 0.50 mg/day (range 0.44−;0.97).

All patients (N=68) reported at least one AE and 28/68 (41.2%) patients had a grade 3/4 AE (**Table 2**). The most common AEs (≥30%) were hyperglycemia and nausea (51.5% each), diarrhea (44.1%) and cholelithiasis (33.8%). Treatment-related AEs (TRAEs) were reported in 66/68 (97.1%) patients; the most frequent TRAEs (≥30%) were hyperglycemia and nausea (47.1% each), diarrhea (39.7%), and cholelithiasis (32.4%). Fourteen (20.6%) patients had ≥1 AE leading to discontinuation.

**Table 2:** Summary of adverse events (≥10%), overall and by treatment regimen.

	All patients N=68 Any grade n (%)	Grade 3/4 N=68 n (%)	Pasireotide monotherapy^†^ n=42 n (%)	Grade 3/4 n=42 n (%)	Combination therapy (after the addition of cabergoline) n=42 n (%)	Grade 3/4 n=42 n (%)
Any AE*	68 (100)	28 (41.2)	39 (92.9)	13 (31.0)	39 (92.9)	10 (23.8)
Hyperglycemia^a^	35 (51.5)	5 (7.4)	21 (50.0)	4 (9.5)	7 (16.7)	0
Nausea	35 (51.5)	0	16 (38.1)	0	7 (16.7)	0
Diarrhea	30 (44.1)	1 (1.5)	22 (52.4)	1 (2.4)	6 (14.3)	0
Cholelithiasis	23 (33.8)	2 (2.9)	4 (9.5)	0	12 (28.6)	1 (2.4)
Headache	20 (29.4)	2 (2.9)	11 (26.2)	0	2 (4.8)	1 (2.4)
Dizziness	19 (27.9)	0	9 (21.4)	0	5 (11.9)	0
Back pain	13 (19.1)	0	4 (9.5)	0	4 (9.5)	0
Fatigue	13 (19.1)	0	9 (21.4)	0	2 (4.8)	0
Abdominal pain	12 (17.6)	0	7 (16.7)	0	3 (7.1)	0
Hypoglycemia	12 (17.6)	2 (2.9)	2 (4.8)	0	5 (11.9)	0
GGT increased	11 (16.2)	3 (4.4)	7 (16.7)	0	5 (11.9)	2 (4.8)
Nasopharyngitis	11 (16.2)	0	4 (9.5)	0	5 (11.9)	0
Alopecia	10 (14.7)	0	3 (7.1)	0	5 (11.9)	0
Pain in extremity	9 (13.2)	0	6 (14.3)	0	1 (2.4)	0
Pruritis	9 (13.2)	0	3 (7.1)	0	1 (2.4)	0
Depression	8 (11.8)	1 (1.5)	5 (11.9)	1 (2.4)	1 (2.4)	0
Rash	8 (11.8)	0	1 (2.4)	0	2 (4.8)	0
Anemia	7 (10.3)	1 (1.5)	1 (2.4)	0	2 (4.8)	1 (2.4)
Asthenia	7 (10.3)	0	1 (2.4)	0	2 (4.8)	0
Insomnia	7 (10.3)	0	3 (7.1)	0	1 (2.4)	0

*Excluding hematological and biological AEs.

^†^Data shown relate to AEs reported during pasireotide monotherapy for the 42 patients who received cabergoline.

a Hyperglycemia is self-monitored fasting blood glucose value of >130 mg/dL AE, adverse event; GGT, gamma-glutamyl transferase.

The most common AEs leading to discontinuation were increased gamma-glutamyl transferase (GGT) and hyperglycemia (two patients each, 2.9%). Twenty-three (33.8%) patients had ≥1 AE leading to dose adjustment or interruption. Details on special safety assessments such as hyperglycemia-related AEs, blood glucose, HbA1c, IGF-1 as well as hematological and biochemical abnormalities are presented in the **Supplementary Appendix**.

Three (4.4%) patients died during the study, two (2.9%) during the core phase and one (1.5%) during the extension. All deaths were considered unrelated to study medication. The causes during the core phase were multi-organ dysfunction syndrome for one patient aged 79 years and unknown for the other aged 34 years. Uncontrolled hypertension was reported as the cause of death for the patient aged 47 during the extension phase.

## 4 Discussion

The severe morbidity and increased mortality with uncontrolled CD highlight the importance of identifying an effective medical strategy. This study explored the potential of a synergistic benefit of the addition of cabergoline to pasireotide treatment in patients with CD.

Complete normalization of cortisol production is required to reverse the risks of morbidity and mortality in patients with CD (1). Two small studies showed clinical improvement of normalized UFC when cabergoline and ketoconazole were combined (20, 21). Benefit has also been reported with triple therapy with pasireotide, cabergoline and ketoconazole (18) and triple therapy with ketoconazole, metyrapone and mitotane in severe CD (22). In the current study, 50% of patients achieved the primary endpoint of mUFC ≤ULN at week 35 and a similar proportion (48%) sustained biochemical control throughout the extension phase. Notably, combination treatment doubled the number of patients who attained mUFC ≤ULN from the core phase to the end of the extension phase. In particular, mUFC was rapidly reduced with treatment, i.e., in most patients within 2 months, while measures of patient-reported outcomes also improved including QoL. Twenty-three patients (33.8%) who completed the core phase did not enter the extension phase. This was because only patients from countries where a commercial supply was unavailable were given the option to enter the extension phase.

This study confirms previous reports that patients with mild hypercortisolism at baseline were more likely to achieve mUFC control with pasireotide monotherapy than patients with moderate or severe hypercortisolism (6, 23). In addition, patients with moderate hypercortisolism at baseline were more likely to achieve mUFC control with the addition of cabergoline. This supports that a combination therapy can be effective for patients with a wider range of disease severity. Accordingly, *in vitro* data may indeed indicate synergism between SSTR and D2 that might increase therapeutic efficacy (24, 25).

Improvements in clinical signs and symptoms with pasireotide monotherapy were consistent with published data (6, 10). In the core phase, an improvement of blood pressure and BMI was observed with pasireotide monotherapy and, to a lesser extent, with combination therapy which may related to the difference in duration of biochemical remission.

The overall safety profile was consistent with that expected for pasireotide, with most AEs being mild/moderate (26, 27). There were no new safety signals identified with the addition of cabergoline. Common AEs including nausea, headache, dizziness, and fatigue are suggestive of steroid withdrawal symptoms associated with the decrease in UFC, although direct drug effects cannot fully be excluded. Adrenal insufficiency was not reported as side effect. Rates of hyperglycemia-related AEs (68%) were consistent with those in previous reports of pasireotide monotherapy (6, 10). FPG increased with pasireotide monotherapy during the first 8 weeks of treatment and stabilized for the remainder of the study, including following the addition of cabergoline. These data highlight the vital role of blood glucose monitoring in these patients.

Both pasireotide and cabergoline are pituitary-targeted agents that act directly on the source of the disease via inhibition of ACTH release by the corticotroph tumor, which may be an advantage over steroid synthesis inhibitors. This study further confirms previous data reporting the benefits of pasireotide in combination with cabergoline in patients with CD (18). While not entirely elucidated, down-regulation of dopamine D2 receptors (D2R) expression, and post-receptor desensitization and/or tumor regrowth of corticotroph tumor cell were suggested as possible mechanisms for treatment escape (15). Moreover, different dopamine receptor patterns and/or D2R isoforms also influence the response and eventually the treatment escape. Treatment escape has been observed in some studies after long-term (7−;12 months) treatment with cabergoline (13), however it is possible that use of concomitant SRLs could potentially reduce the rate of escape. In this study, a total of 13 patients experienced treatment escape. However, 10 of these patients regained biochemical control. For 7 of these 10 patients, there was up titration of doses to a maximum of 1.8 mg/day of pasireotide and 1 mg/day of cabergolineAlthough pasireotide and cabergoline have shown long-term reduction in IGF-1 levels in patients with acromegaly (28, 29), there is little evidence for this effect in patients with CD (4, 30). One study (n=17) found significant decreases in IGF-1 after 28 days’ treatment with pasireotide that was independent of UFC reduction. One-third of patients had low IGF-1 (30). Our study showed that almost half of patients (47.6%) had IGF-1 levels either above ULN or below LLN prior to the addition of cabergoline, and IGF-1 levels decreased relative to the baseline, with majority of values within the normal range during the core and extension phases up to week 99. Baseline levels of IGF-1 may already be low because of the suppressive effect of excess cortisol on the somatotropic axis (31).

Although clinicians have several therapeutic options at their disposal to treat hypercortisolemia associated with CD, the optimal treatment approach should be based on the individual clinical situation and the benefit–risk considerations for each patient. In this study, 13 patients had history of pituitary radiation, with a duration of at least 2.6 years (median 3.3 years) between the last radiation treatment and the observed response date. However, only 7/13 patients achieved the therapeutic target. Although there was a gap of > 2 years, we cannot exclude the role of radiation in normalizing UFC. Contrastingly, 6/13 patients treated with radiation did not achieve mUFC ≤ULN (responders) at Week 35. The impact of the adjuvant radiation therapy remains unclear.

The strengths of this study are that this is the largest and longest prospective study with pituitary-directed pharmacotherapy, to date, evaluating the addition of cabergoline to pasireotide in patients with CD, and this stepwise approach reflects real-world clinical practice (18). The study is limited by the open-label design and the fact that it was not a head-to-head comparative study of pasireotide only versus pasireotide plus cabergoline. This may be of importance in interpreting patient-reported outcomes. Several patients continued treatment for almost 2 years; however, interpretation of long-term data should be made with caution because of the small patient numbers. Notably, the last available assessment was carried forward for patients with missing mUFC value at week 35 including those who discontinued and were considered for response analysis. It should also be noted that the definition of loss of response, also known as escape, used in this study (at least one UFC value >ULN after previously achieving UFC ≤ULN) may overestimate the rate of apparent escape as UFC values may have fluctuated about the ULN range or been marginally elevated. The definition of treatment escape differs across studies, and we have used a very stringent one in this study, requiring only a single high UFC to meet the classification as escape. Thus, it is likely that some loss of biochemical control interpreted as escape is actually fluctuation of cortisol around the upper limit of normal range.

Other limitations include protocol deviations in including 3 patients with normal UFC at baseline (one patient was uncontrolled at rescreen, and one was discontinued at 2 weeks - both classified as

non-responders), lack of data on impact of radiation therapy without study drug in patients who gained biochemical control with adjuvant radiation therapy, lack of pituitary magnetic resonance imaging to detect pituitary tumor changes, lack of data about effective cabergoline dose and absence of cardiac valve assessment for mild to moderate severity in the medium term. Both pasireotide and cabergoline can induce tumor shrinkage in CD (6, 9, 32–35) and it would be interesting to examine the combined effect on tumor size. This study used the subcutaneous formulation of pasireotide, whereas the most common usage currently is the long-acting formulation. Efficacy of long-acting pasireotide (36) seems higher compared to the subcutaneous formulation (7) and the effect of combination of long-acting pasireotide with cabergoline should be evaluated in future studies. No formal assessments were made for impulsive control disorders, which have been associated with dopamine agonists, including cabergoline (32, 33, 37, 38). The reason that several different terms were used for hyperglycemia-related AEs is that they were reported as per discretion of each investigator. No additional psychiatric AEs were reported, although they were not exhaustively searched.

## 5 Conclusions

This is the first study demonstrating that pituitary-targeted combination treatment with pasireotide and cabergoline doubled the number of patients who attained mUFC ≤ULN. Both short- and long-term safety profile are consistent with known data for pasireotide and cabergoline. The low rate of discontinuation due to AEs suggests that pasireotide alone or as combination treatment is generally well-tolerated if appropriately monitored, even with prolonged treatment. The addition of cabergoline to pasireotide treatment in patients with persistently elevated mUFC could be an effective long-term strategy for enhancing the control of CD in a subset of patients, with close monitoring for possible escape.

## Data availability statement

The original contributions presented in the study are included in the article/**Supplementary Material**. Further inquiries can be directed to the corresponding author.

## Ethics statement

The studies involving humans were approved by Hospital Britanico, Buenos Aires, Argentina; Ethische commissie University Hospitals Leuven, Leuven, Belgium; Universitair Ziekenhuis Gent, Gent, Belgium; Comite de Etica em Pesquisa Hospital Moinhos de Vento, Porto Alegre-RS, Brazil; Comitê de Ética em Pesquisa do Hospital de Clínicas, Universidade Federal do Paraná, Curitiba-PR, Brazil; Comissão de Ética para Análise de Projetos de Pesquisa, São Paulo - SP, Brazil; Ethics Committee for clinical trials, Sofia, Bulgaria; Comité Corporativo de Ética en Investigación, Bogotá DC, Colombia; Comite De Protection Des Personnes, Groupe Hospitalier Pellegrin - Bat, Bordeaux Cedex, France; Friedrich-Alexander Universitat Erlangen-Nurnberg, Medizinische Fakultat, Erlangen, Germany;National Ethics Committee, Cholargos, Athens, Greece; Ethics Committee for Clinical Pharmacology (ECCP), Budapest, Hungary; Institute Ethics Committee, New Delhi, India; Institutional Review Board (IRB) Ethics Committee Silver, Christian Medical College, Vellore, Tamil Nadu, India; Institute Ethics Committee, PGIMER, Chandigarh, India; Comitato Etico Dell’irccs Istituto Auxologico Italiano Di Milano, Milano, Italy; Comitato Etico Universita’ Federico Ii Di Napoli, Napoli, Italy; Jawatankuasa Etika & Penyelidikan Perubatan (Medical Research and Ethics Committee), d/a Institut Pengurusan Keshatan Jalan Rumah Sakit, Kuala Lumpur, Malaysia; Institutd Nacional De Neurologia Y Neurocirugia, Mexico City, Mexico; Clinica Bajio (CLINBA), Guanajuato, Mexico; Medische Ethische Toetsings Commissie, Rotterdam; Netherlands; CEIm Provincial de Málaga, Málaga, Spain; Istanbul University Cerrahpasa Medical Faculty, Istanbul, Turkey; WIRB, Puyallup, WA, USA; Research Integrity Office, Oregon Health & Science University Portland, OR USA. The studies were conducted in accordance with local legislations and institutional requirements. The participants provided their written informed consent to participate in this study.

## Author contributions

All authors directly participated in the planning, execution, or analysis, and have had full control of complete primary data, and hold responsibility for data integrity and accuracy. All authors contributed to the article and approved the submitted version.
