# Supplementary material for: Long-term efficacy and safety of subcutaneous pasireotide alone or in combination with cabergoline in Cushing’s disease
Source: Front Endocrinol (Lausanne). 2023 Oct 9;14:1165681. doi: 10.3389/fendo.2023.1165681 (PMC10593462; doi:10.3389/fendo.2023.1165681)
Supplement: Supplementary file 1 [file DataSheet_1.docx]

**Supplementary Appendix**

Inclusion Criteria

Adult patients with confirmed diagnosis of ACTH-dependent Cushing’s disease as evidenced by all of the following: 1) the mean of three 24-hour urine samples collected within 2 weeks above ULN, 2) morning plasma ACTH within the normal or above normal range and 3) either MRI confirmation of pituitary adenoma > 6 mm, or inferior petrosal sinus (IPSS) gradient >3 after CRH stimulation (or >2 if IPSS was previously performed without CRH stimulation) for those patients with a tumor ≤6 mm. If IPSS had not previously been performed, IPSS with CRH stimulation is required. For patients who had prior pituitary surgery, histopathology confirming an ACTH staining adenoma was considered confirmatory of CD. Patients with de novo CD were eligible if they were poor surgical candidates, had surgically unapproachable tumors, or refused surgery. Candidates could be pasireotide naïve at screening, have previously received pasireotide and discontinued the drug for reasons other than safety, or be uncontrolled on the maximum tolerated dose of pasireotide. The washout period for patients who were previously treated with pasireotide was two weeks for subcutaneous and 12 weeks for long-acting release regimens; 1 week for steroidogenesis inhibitors and 4 weeks for dopamine agonists.

Exclusion Criteria

Patients with compression of the optic chiasm causing any visual field defect that requires surgical intervention. Diabetic patients with poor glycemic control as evidenced by HbA1c >8%. Patients with risk factors for torsade de pointes, i.e. patients with a baseline QTcF >450 ms in males, and > 460 ms in females. Hypokalemia, hypomagnesaemia, uncontrolled hypothyroidism, family history of long QT syndrome, or concomitant medications known to prolong QT interval. Patients with clinically significant valvular disease. Patients with Cushing’s syndrome due to ectopic ACTH secretion. Patients with hypercortisolism secondary to adrenal tumors or nodular (primary) bilateral adrenal hyperplasia. Patients who have a known inherited syndrome as the cause for hormone over-secretion (i.e., Carney Complex, McCune-Albright syndrome, MEN-1). Patients who are hypothyroid and not on adequate replacement therapy. Patients with symptomatic cholelithiasis. Patients who have congestive heart failure (NYHA Class III or IV), unstable angina, sustained ventricular tachycardia, clinically significant bradycardia, advanced heart block, history of acute MI less than one year prior to study entry or clinically significant impairment in cardiovascular function. Patients with liver disease such as cirrhosis, chronic active hepatitis, or chronic persistent hepatitis, or patients with ALT/AST > 2 X ULN, serum bilirubin >2.0 X ULN. Patients with serum creatinine >2.0 X ULN. Patients with WBC <3 X 10^9^/L; Hb 90% < LLN; PLT <100 X 10^9^/L. Patients who have a history of alcohol or drug abuse in the 6-month period prior to receiving pasireotide. Patients who have participated in any clinical investigation with an investigational drug within 1 month prior to dosing. Patients with active malignant disease within the last five years (with the exception of basal cell carcinoma or carcinoma in situ of the cervix). Patients with the presence of active or suspected acute or chronic uncontrolled infection. Patients with a history of non-compliance to medical regimens or who are considered potentially unreliable or will be unable to complete the entire study. Patients with presence of Hepatitis B surface antigen (HbsAg). Patients with presence of Hepatitis C antibody test (anti-HCV). Patients with severe hepatic impairment (Child Pugh C) and hypersensitivity to pasireotide or cabergoline. Patients with lung, pericardial, and retroperitoneal fibrosis; gastro-duodenal ulcer or digestive hemorrhage, galactose intolerance, Parkinson’s disease, uncontrolled hypertension and Raynaud’s syndrome. Pregnant or nursing (lactating) women where pregnancy is defined as the state of a female after conception and until the termination of gestation, confirmed by a positive hCG laboratory test (> 5 mIU/mL). Patients with end-stage renal failure and/or hemodialysis.

End Points and Assessments

Safety and tolerability (including adverse events [AEs], fasting plasma glucose [FPG], HbA_1c_, insulin-like growth factor 1 [IGF-1]) were monitored throughout the study and assessed at the end of the core and extension phases. National Cancer Institute Common Terminology Criteria for Adverse Events version 4.0 was used to assess safety and clinical laboratory tests (hematology, biochemistry, urinalysis).

Statistical Analyses

*Post hoc* analyses were conducted to determine the effect of adding cabergoline to pasireotide monotherapy in the subset of patients who received combination therapy during the core or extension phase on mUFC, serum cortisol, clinical signs and symptoms, and safety (AEs, IGF-1, FPG). Severity of hypercortisolism was defined as follows: mild, mUFC 1.0–<2.0 x ULN; moderate, mUFC 2.0–≤5.0 x ULN; severe, mUFC >5.0 x ULN.

Sample Size Estimation

Sample size was based on the estimated proportion of patients achieving normalized mUFC at week 35 among those who were pasireotide naïve or treated with pasireotide but still with uncontrolled mUFC at screening. The sample size of 64 patients was based on the assumption that 34% of patients would achieve mUFC ≤ULN at week 35 and the precision of the response would be 12.8% for the associated two-sided 95% confidence interval (CI), with a dropout rate of 10%.

Special Safety Assessments

Hyperglycemia-related AEs were reported in 46 (67.6%) patients; six (8.8%) patients had grade 3/4 AE related to hyperglycemia. Four patients (5.9%) required dose adjustment and two (2.9%) discontinued treatment. There was one hyperglycemia-related serious AE (1.5%). The proportion of patients reporting hyperglycemia, diabetes mellitus or impairment of glucose tolerance was lower following the addition of cabergoline to pasireotide than during treatment with pasireotide alone. Half of patients who received pasireotide monotherapy prior to the addition of cabergoline reported hyperglycemia events (50%; 21/42), with 16.7% (7/42) doing so after the addition of cabergoline.

During the first 8 weeks, there was an increase in mean FPG from 5.3±1.23 to 7.1±2.67 mmol/L and HbA_1c_ from 5.9% to 7.2%, and then remained stable for the remainder of the study (Supplementary Figures 2a and 2b). Patients who received pasireotide monotherapy for the duration of the study had lower FPG than those receiving combination therapy up to week 99. Six of 68 (8.8%) patients took metformin prior to study entry. During the study, 22 (32.4%) patients required metformin. In sixteen (23.5%) patients, metformin prescribed newly.

Overall, IGF-1 levels decreased relative to baseline (median 19.3 nmol/L; range 4‒49; n=68) in both the core (week 35: median 9.6 nmol/L; range 4‒27; n=39) and extension phases (week 99: median 10.1 nmol/L; range 4‒30; n=16). Prior to the addition of cabergoline, 20/42 (47.6%) patients had IGF-1 levels within the abnormal range (values below or above lower limit of normal (LLN) [10.06 nmol/L] or ULN [35.49 nmol/L], respectively, were considered abnormal). During the first 8 weeks after the addition of cabergoline, the proportion of patients with abnormal levels of IGF-1 increased (14/23; 60.9%). At weeks 35, 43 and 99, 3/9 (33.3%), 3/6 (50.0%) and 1/3 (33.3%) patients had abnormal IGF-1 levels, respectively. QT-prolongation-related AEs were infrequent (reported in 2.9% of patients).

There were no grade 3/4 hematological abnormalities during the core phase. The most frequent (≥4%) grade 3/4 biochemical abnormalities included elevations in glucose (12.1%), uric acid (6.1%), triglycerides (4.5%), potassium (4.5%), lipase (blood; 4.5%), and GGT (4.5%) in the core phase. In the extension phase, 1/42 (2.4%) patient who received combination therapy had grade 3/4 anemia. The most frequent (≥4%) grade 3/4 biochemical abnormalities in the extension phase were elevated GGT (4.8%) and alanine aminotransferase (4.8%).

Supplementary Figure S1. Study treatment schedule

^1^Patients not achieving normal UFC at the end of each treatment period had the dose of the medication up titrated to the next level; ^2^If biochemical control was not achieved, patients received at least 8 weeks of study treatment at each dose level before being up titrated to the next dose level; ^3^Patients who could not have the dose up titrated to 0.9 mg for safety reasons (e.g. increased blood glucose levels) were treated with the combination therapy (pasireotide 0.6 mg and cabergoline at increasing doses). Patients who could not tolerate 0.6 mg bid had the dose down titrated to 0.3 mg bid

Supplementary Figure S2. (a) Actual mean changes in FPG overall and by treatment regimen and (b) Actual mean changes in HbA1c overall

^†^Number of patients with values recorded at each time window

FPG, fasting plasma glucose; hbA1c, glycated hemoglobin

Supplementary Figure S3. Patients with improvement in clinical signs of Cushing’s disease relative to baseline

^†^Number of patients with values recorded at each time window

**Supplementary Table S1. Clinical signs of hypercortisolism over time**

| **Median (range)** | **All patients**  **N=68** | **Pasireotide monotherapy** | **Combination therapy (before the addition of cabergoline)** | **Combination therapy (after the addition of cabergoline)** |
| --- | --- | --- | --- | --- |
| **Systolic blood pressure, mmHg** | | | | |
| Baseline  Week 35**^†^**  Week 99**^†^** | 126.5 (87‒161); n=68  121.0 (83‒163); n=41  117.3 (83‒131); n=16 | 125.7 (87‒161); n=26  112.7 (87‒148); n=13  109.8 (83‒131); n=8 | 127.5 (88‒143); n=40  116.7 (117‒117); n=1  ‒ | 124.7 (85‒161); n=41*  122.0 (83‒163); n=27  118.0 (112‒126); n=7 |
| **Diastolic blood pressure, mmHg** | | | | |
| Baseline  Week 35**^†^**  Week 99**^†^** | 81.3 (59‒104); n=68  79.3 (48‒103); n=41  81.7 (59‒100); n=16 | 82.7 (64‒104); n=26  78.7 (48‒92); n=13  76.2 (59‒90); n=8 | 80.7 (59‒101); n=40  83.3 (83‒83); n=1  ‒ | 79.7 (59‒102); n=41*  79.3 (52‒103); n=27  83.3 (71‒100); n=7 |
| **Body mass index, kg/m^2^** | | | | |
| Baseline  Week 35**^†^**  Week 99**^†^** | 29.5 (19‒51); n=68  27.5 (17‒47); n=41  27.5 (19‒45); n=16 | 29.6 (19‒44); n=26  25.6 (17‒32); n=13  26.6 (19‒33); n=8 | 30.0 (21‒51); n=40  37.0 (37‒37); n=1  ‒ | 28.2 (20‒49); n=41*  28.0 (19‒47); n=27  31.6 (25‒45); n=7 |
| **Total cholesterol, mmol/L** | | | | |
| Baseline  Week 35**^†^**  Week 99**^†^** | 5.3 (3‒9); n=68  5.0 (3‒7); n=41  5.1 (4‒9); n=16 | 5.2 (3‒8); n=26  5.0 (3‒7); n=13  5.1 (4‒9); n=8 | 5.3 (4­‒9); n=42  5.5 (5‒5); n=1  ‒ | 4.8 (3‒7); n=41*  5.0 (3‒7); n=27  4.8 (4‒6); n=7 |

**^†^Number of patients with values recorded at each time window***Start of cabergoline

SD, standard deviation

Supplementary Table S2. CushingQoL standardized score up to week 35

| **Week** | **Actual mean score (SD)** | **Mean percentage change (SD)** |
| --- | --- | --- |
| Baseline (N=68) | 41.6 (20.2) |  |
| Week 35 (n=40) | 47.6 (20.8) | 69.5% (279.9%) |

QoL, quality of life; SD, standard deviation

### **Supplementary Figure S1. Study treatment schedule**


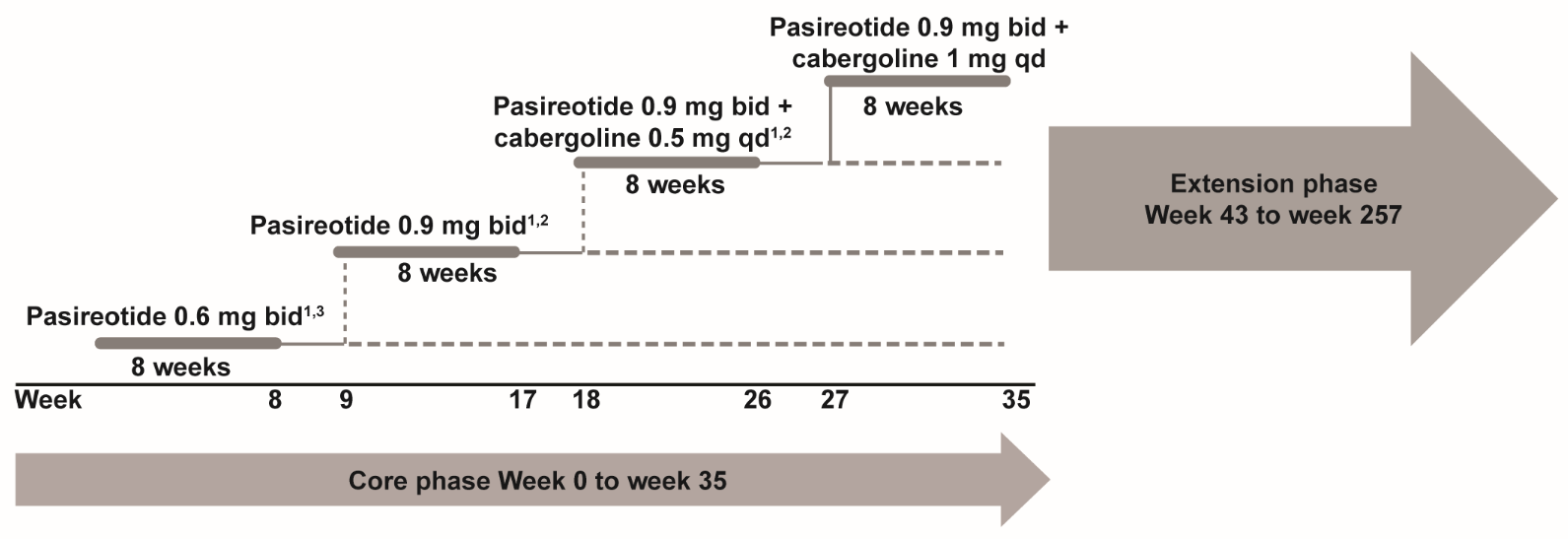


^1^Patients not achieving normal UFC at the end of each treatment period had the dose of the medication uptitrated to the next level; ^2^If biochemical control was not achieved, patients received at least 8 weeks of study treatment at each dose level before being uptitrated to the next dose level; ^3^Patients who could not have the dose uptitrated to 0.9 mg for safety reasons (e.g. increased blood glucose levels) were treated with the combination therapy (pasireotide 0.6 mg and cabergoline at increasing doses). Patients who could not tolerate 0.6 mg bid had the dose downtitrated to 0.3 mg bid

### **Supplementary Figure S2. (a) Actual mean changes in FPG overall and by treatment regimen and (b) actual mean changes in HbA1c overall**^†^


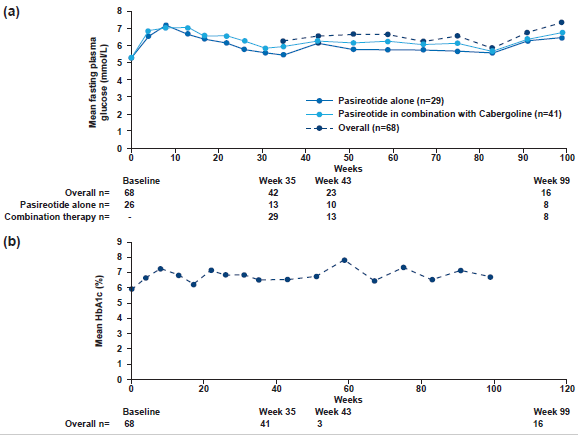


^†^Number of patients with values recorded at each time window

### **Supplementary Figure S3. Patients with improvement in clinical signs of Cushing’s disease relative to baseline**^†^


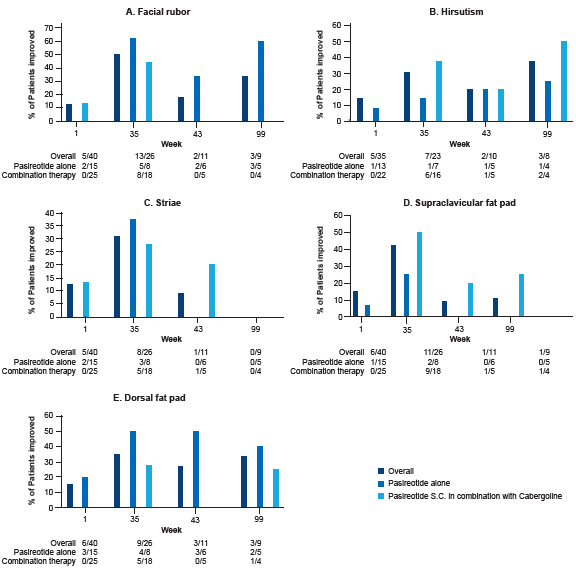


^†^Number of patients with values recorded at each time window
